# Obesity Control and Supplementary Nutraceuticals as Cofactors of Brain Plasticity in Multiple Sclerosis Populations

**DOI:** 10.3390/ijms252010909

**Published:** 2024-10-10

**Authors:** Lorena Ciumărnean, Oliviu-Florențiu Sârb, Nicu-Cătălin Drăghici, Octavia Sălăgean, Mircea-Vasile Milaciu, Olga-Hilda Orășan, Călin-Vasile Vlad, Irina-Maria Vlad, Teodora Alexescu, Ioana Para, Simina-Felicia Țărmure, Elisabeta-Ioana Hirișcău, Gabriela-Bombonica Dogaru

**Affiliations:** 1 Department of Internal Medicine, “Iuliu Hațieganu” University of Medicine and Pharmacy, 400015 Cluj-Napoca, Romania; lorena_ciumarnean@yahoo.com (L.C.); mircea_milaciu@yahoo.com (M.-V.M.); olgaorasan@yahoo.com (O.-H.O.); vladvasilecalincfcluj@gmail.com (C.-V.V.); teodora.alexescu@gmail.com (T.A.); ioana.para@yahoo.com (I.P.); siminatarmure@yahoo.com (S.-F.Ț.); 2 Department of Clinical Neurosciences, “Iuliu Hațieganu” University of Medicine and Pharmacy, 400012 Cluj-Napoca, Romaniairina.vlad001@gmail.com (I.-M.V.); 3 “IMOGEN” Institute, Centre of Advanced Research Studies, Emergency Clinical County Hospital Cluj, 400347 Cluj-Napoca, Romania; 4 Department of Nursing, Faculty of Medicine, “Iuliu Hațieganu” University of Medicine and Pharmacy, 400012 Cluj-Napoca, Romania; runcan.octavia@yahoo.com (O.S.); ioanahiriscau@gmail.com (E.-I.H.); 5 Department of Medical Rehabilitation, “Iuliu Hațieganu” University of Medicine and Pharmacy, 400012 Cluj-Napoca, Romania

**Keywords:** multiple sclerosis, neurodegeneration, neurorehabilitation, nutraceuticals, obesity

## Abstract

Multiple sclerosis (MS) is an immune-mediated disease characterized by inflammation, demyelination, and neurodegeneration within the central nervous system. Brain plasticity, the brain’s ability to adapt its structure and function, plays a crucial role in mitigating MS’s impact. This paper explores the potential benefits of lifestyle changes and nutraceuticals on brain plasticity in the MS population. Lifestyle modifications, including physical activity and dietary adjustments, can enhance brain plasticity by upregulating neurotrophic factors, promoting synaptogenesis, and reducing oxidative stress. Nutraceuticals, such as vitamin D, omega-3 fatty acids, and antioxidants like alpha lipoic acid, have shown promise in supporting brain health through anti-inflammatory and neuroprotective mechanisms. Regular physical activity has been linked to increased levels of brain-derived neurotrophic factor and improved cognitive function. Dietary interventions, including caloric restriction and the intake of polyphenols, can also positively influence brain plasticity. Integrating these lifestyle changes and nutraceuticals into the management of MS can provide a complementary approach to traditional therapies, potentially improving neurological outcomes and enhancing the quality of life for the MS population.

## 1. Introduction

Multiple sclerosis (MS) is the most common immune-mediated disease affecting the central nervous system (CNS), characterized by neuronal degeneration due to inflammation, demyelination, and axonal damage [1,2]. Lesions in MS involve multifocal demyelination, leading to oligodendrocyte destruction and glial scars, with axonal injury occurring later in the disease course [3]. Common symptoms include visual disturbances, paresthesia, urinary and intestinal incontinence, focal weakness, and cognitive dysfunction, particularly in young adults during acute relapses [4].

MS immunopathology involves complex interactions between T and B cells. T lymphocytes, particularly CD4+ (Th1 and Th17) and CD8+ cells, play key roles, while regulatory T cells (Tregs) may offer protection [1,2,3,4]. B cells contribute through antigen presentation, cytokine secretion, and antibody production, with ectopic B cell follicles linked to severe cortical pathology in progressive MS [1,2,3,4]. Anti-CD20 therapies have highlighted the significance of B cells in MS pathogenesis, and treatments targeting both B and T cells show promise [1,2,3,4].

MS is classified into stages: relapsing–remitting MS (RRMS), primary progressive MS (PPMS), secondary progressive MS (SPMS), MS with progressive relapses, isolated clinical syndrome, fulminant MS, and benign MS [1,3,5]. RRMS is the most common form, where relapses are followed by partial or complete recovery, though residual symptoms can contribute to long-term disability [1,3,5]. The McDonald criteria are widely used for diagnosis, focusing on dissemination in space (DIS) and time (DIT), with MRI and cerebrospinal fluid (CSF) analysis aiding early detection, particularly through the presence of oligoclonal bands (OCBs) [6,7].

Long-term treatment aims to reduce relapse rates and prevent permanent disability [7].

## 2. Risk Factors for the Development and Progression of MS

The exact cause of MS remains unknown, but several risk factors have been identified [8]. Studies suggest that individuals with autoimmune diseases, such as type 1 diabetes, have double the risk of developing MS compared to the general population [8,9,10].

Genetic susceptibility is significant, with over 200 polymorphisms associated with MS identified, though the mechanism remains unclear. These polymorphisms are often found in regulatory regions of immune-related genes and are linked to other autoimmune conditions [11,12,13].

External factors like viral infections, geographic location, sunlight exposure, vitamin D3 levels, smoking, obesity, and vaccination status may also influence MS risk [14,15]. Epstein–Barr virus (EBV) is particularly implicated, possibly triggering immune responses that lead to MS onset, though other viruses may play a role as well [16,17]. Sunlight, through UV exposure or by increasing vitamin D3 levels, offers some protection, correlating MS risk with latitude and altitude [18]. Smoking is another risk factor, possibly due to oxidative stress from tobacco byproducts, although the exact mechanism is unclear [19,20].

Understanding MS risk factors is crucial for the following:

1. Prevention and early intervention: Addressing modifiable factors like vitamin D deficiency, smoking, and obesity may reduce MS risk [18,20].
2. Improved counseling: Health professionals can provide better guidance to high-risk individuals, including relatives of MS patients [1,2,3,4,5,6].
3. Personalized care: Tailoring treatment based on individual risk profiles may slow disease progression [3,5].
4. Research: Identifying risk factors helps guide studies on MS pathogenesis and treatment [12].
5. Gene–environment interactions: Understanding how environmental factors interact with genetics offers insights into disease mechanisms [11,12,13].
6. Public health: Risk factor knowledge can inform public health strategies [19,20].
7. Prognosis: Some risk factors may affect disease progression, offering valuable prognostic information [11,12,13,14,15,16,17,18,19,20].

Addressing these risk factors could help reduce MS incidence and improve outcomes while advancing our understanding of the disease.

## 3. Brain Plasticity

Brain plasticity refers to the brain’s ability to change its structure and function in response to experiences, learning, and environmental stimuli [21]. It is a fundamental process that allows the brain to adapt, learn, and recover from injuries or diseases [22]. Plasticity can occur at the cellular and molecular levels, leading to changes in neuronal properties, synaptic connections, and neural networks [23]. Research has shown that brain plasticity is present throughout the lifespan, although the efficiency of plasticity may decline with age [24]. Genetic factors also play a role in brain plasticity, with heritability estimates suggesting that genetic variations contribute to interindividual variability in plasticity [25].

**Figure 1:**
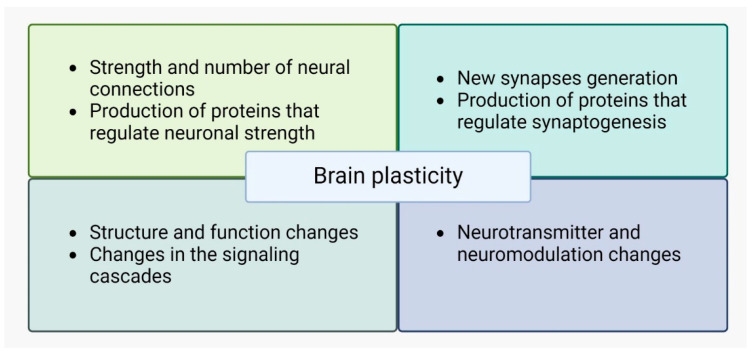
Mechanisms involved in brain plasticity.

Brain plasticity refers to the brain’s ability to reorganize and modify neural connections in response to experience, learning, injury, and disease. This involves modifications in synaptic strength and number through long-term potentiation and long-term depression, which are activity-dependent changes. Additionally, plasticity encompasses synaptogenesis, the creation of new synapses that rewire neural circuits, as well as alterations in neuron structure and function, including dendritic branching and neurogenesis. Changes in neurotransmitter and neuromodulator levels also influence synaptic transmission and neuronal excitability, while complex signaling cascades activate genes and produce proteins that regulate synaptic plasticity and connectivity [26,27,28] (Figure 1).

Brain plasticity has important implications in various contexts, including neurodevelopmental disorders, addiction, stroke recovery, and neurodegenerative diseases [22,29,30].

Physical activity has a significant impact on brain plasticity, which refers to the brain’s ability to reorganize and modify its neural connections. Several studies have shown that physical activity can enhance brain plasticity and promote various neuroadaptive processes.

One study found that voluntary exercise in rats increased both precursor and mature forms of brain-derived neurotrophic factor (BDNF) in the hippocampus, a region important for learning and memory [31]. BDNF is a neurotrophic factor that plays a crucial role in synaptic plasticity and neuronal survival. Exercise also increased the activity of tissue-type plasminogen activator (tPA), an enzyme involved in the cleavage of pro-BDNF into mature BDNF. Blocking tPA activity reduced the exercise-induced effects on BDNF and its downstream signaling pathways [31].

Regular physical activity has also been shown to enhance brain plasticity in humans. Studies have demonstrated that aerobic exercise, anaerobic exercise, and resistance exercise can positively influence brain plasticity and cognitive function [32]. These exercise modalities can lead to changes in the expression of neurotrophic factors, such as BDNF, lactate, and vascular endothelial growth factor, which promote neurogenesis and synaptic plasticity [32].

Furthermore, physical activity can reduce short-interval intracortical inhibition, a measure of cortical inhibition, and create a more optimal environment for plasticity [33]. Exercise-induced reductions in SICI may contribute to enhanced neuroplasticity and improved motor learning [33].

Overall, physical activity has been shown to promote brain plasticity through various mechanisms, including the upregulation of neurotrophic factors, the modulation of synaptic activity, and a reduction in cortical inhibition. These findings highlight the importance of regular physical activity for maintaining and enhancing brain health and function [31,32,33,34,35,36].

There is limited information available specifically addressing the adverse effects of MS medications on brain plasticity. However, some studies suggest that certain medications used to treat MS may have a positive impact on brain plasticity by reducing inflammation and promoting recovery. One study found that treatment with interferon beta-1a, a commonly used MS medication, improved cortical function and cognitive deficits in newly diagnosed MS populations with gadolinium-enhancing lesions [37]. Another study showed that the reduction in inflammation with interferon beta was associated with the restoration of brain plasticity in MS populations, as evidenced by improvements in task performance and synaptic plasticity [38].

It is important to note that the studies mentioned above focused on the positive effects of MS medications on brain plasticity, rather than adverse effects. Adverse effects of MS medications can vary depending on the specific medication and individual risk factors.

Overall, while there is limited research specifically addressing adverse effects, current evidence suggests that few MS medications may have a positive impact on brain plasticity by reducing inflammation and promoting recovery.

Different diets can have an impact on brain plasticity, which refers to the brain’s ability to change and adapt in response to experiences and environmental factors. Several studies have investigated the effects of various dietary interventions on brain plasticity markers and cognitive function.

Caloric restriction and intermittent fasting have been shown to enhance brain plasticity in animal studies. These dietary interventions have been associated with an increased expression of neurotrophic factors, synaptic function, and adult neurogenesis in the hippocampus, a brain region important for learning and memory [39].

Dietary supplementation with polyphenols and polyunsaturated fatty acids (PUFAs) influences brain plasticity. For instance, docosahexaenoic acid (DHA), an omega-3 fatty acid from fatty fish, affects protein levels related to metabolic homeostasis and synaptic plasticity in the hypothalamus and hippocampus of rats [40], and its supplementation has been linked to improved spatial memory in mice [41]. Moreover, dark chocolate, rich in flavonoids, has demonstrated positive effects on brain functions; in a rat study, various dark chocolate diets reversed chronic isolation stress’s negative impacts on synaptic potency, plasticity, learning, and memory in the hippocampus [42]. These findings indicate that dietary patterns can modulate brain plasticity and cognitive function, though further research is needed to clarify specific mechanisms and optimal dietary interventions for brain health in humans [40,41,42].

## 4. Neurodegeneration in MS

**Figure 2:**
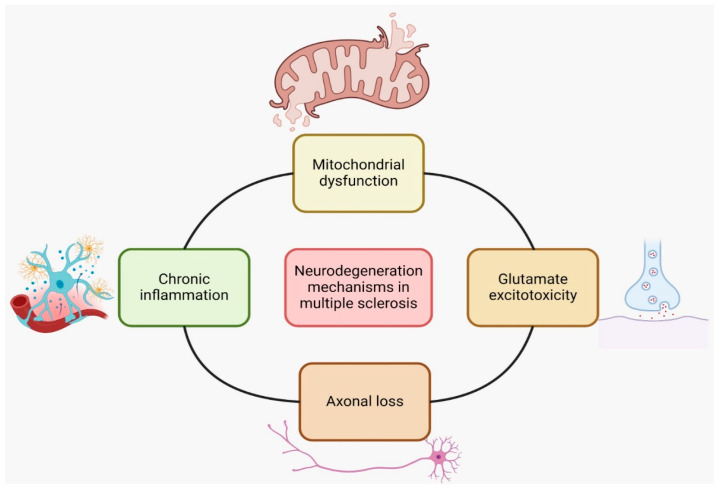
Proposed mechanisms of neurodegeneration in MS.

Neurodegeneration in MS is a key factor in disease progression and disability. Several mechanisms have been proposed to contribute to neurodegeneration in MS, as shown in Figure 2.

1. *Chronic inflammation* in MS leads to the activation of microglia, which releases inflammatory mediators and generates oxidative stress. This can result in damage to neurons and axons [43,44,45]. Neuroinflammation, characterized by the infiltration of immune cells into the central nervous system, can contribute to neurodegeneration in MS. Immune-mediated mechanisms, such as the release of inflammatory cytokines and autoantibodies, can cause neuronal damage [46].
2. *Dysfunction of mitochondria* in axons can lead to energy failure and subsequent neurodegeneration [47,48].
3. *Glutamate*, an excitatory neurotransmitter, can accumulate in the extracellular space during inflammation and demyelination in MS. Excessive glutamate can lead to *excitotoxicity*, causing damage to neurons and axons [45].
4. *Axonal loss* is a major contributor to neurodegeneration in MS. Inflammatory processes, demyelination, and oxidative stress can directly damage axons, leading to their degeneration [49,50].
5. Additional mechanisms include the following:
  - Altered sodium and calcium homeostasis contributes to axonal injury [44].
  - Activated microglia release neurotoxic factors [45].
  - Myelin debris accumulation impairs neurorepair and plasticity.
  - Increased TNF signaling in neurons leads to programmed cell death [50].
  - Impaired astrocytic support may contribute to axonal degeneration [49].
  - Injury to one neuron leads to the degeneration of connected neurons, a process named trans-synaptic degeneration [49,50].

Identifying clinical and lifestyle predictors for MS progression can help target individuals at risk for worsening outcomes. Younger age at onset is associated with a higher risk of progression [51], and men with MS face increased risks compared to women [52]. Those with progressive forms, such as SPMS, are also more likely to experience worsening disease [52]. A greater number of relapses correlates with increased progression risk [52], while higher baseline disability is linked to a greater likelihood of progression [51]. Additionally, smoking is associated with worsening disability [53], whereas higher physical activity levels, both premorbid and current, are linked to a reduced risk of progression [54]. A high-quality diet, particularly one that avoids meat and dairy, is associated with better fatigue and disability outcomes [55], and lower sun exposure during childhood and adolescence increases the risk of disease progression [51].

The most important modifiable factors that act as a risk/protective factor in MS are summarized in Table 1.

Vascular and psychiatric comorbidities have also been associated with subsequent disability worsening in individuals with MS [53].

## 5. Nutraceuticals in MS

Nutraceuticals are natural or bioactive compounds found in food or dietary supplements that have potential health benefits beyond basic nutrition. They are often used as adjunctive treatments to support overall health and well-being.

Several nutraceuticals have been explored for their potential benefits in MS. Vitamin D supplementation, known for its role in immune regulation, has been studied for its ability to reduce disease activity and progression [56,57,58]. Alpha lipoic acid (ALA), an antioxidant, has shown promise in reducing fatigue and improving brain volume [59]. *Ginkgo biloba* has been reported to enhance outcomes like fatigue and antioxidant capacity [59], while biotin supplementation may help reduce disability progression and improve walking ability [59]. Omega-3 fatty acids, with their anti-inflammatory properties, may also help mitigate inflammation in MS [58].

A study found no significant evidence for drug interactions between conventional MS drugs and several nutraceuticals, including ginger, cranberry, vitamin D, fatty acids, turmeric, probiotics, or glucosamine [60].

**Figure 3:**
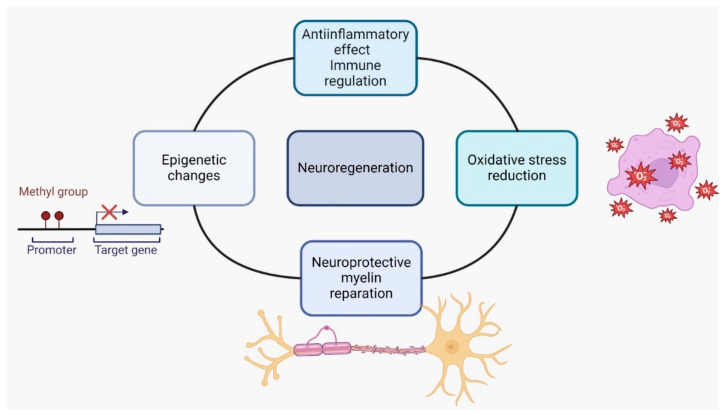
Proposed mechanisms of neurodegeneration through nutraceuticals’ interference.

The presumed mechanisms of action of nutraceuticals in MS involve their ability to target various pathways involved in the pathogenesis of the disease (Figure 3). Some of the proposed mechanisms are as follows:

1. Nutraceuticals such as polyunsaturated fatty acids (PUFAs), green tea flavonoids (epigallocatechin-3-gallate), curcumin, and scorpion toxins have been found to possess anti-inflammatory properties and can *modulate the immune response* in MS. They can inhibit proinflammatory signaling pathways, such as NF-κB or Toll-like receptors and reduce the activity of auto-aggressive immune cells. These effects may help reduce inflammation and immune-mediated damage in MS [56].
2. *Oxidative stress* is implicated in the pathogenesis of MS. Nutraceuticals like green tea, curcumin, and resveratrol have antioxidative properties and can scavenge free radicals, reducing oxidative damage. By reducing oxidative stress, these compounds may protect against neuronal damage and inflammation in MS [61].
3. Nutraceuticals such as flavonoids, terpenoids, and polyphenols have shown potential in promoting *neuroprotection and myelin repair* in animal models of MS. They may support the survival and function of neurons, promote remyelination, and enhance endogenous repair processes [62].
4. Epigenetic modifications play a role in MS. Some nutraceuticals, such as plant polyphenols, Ω-3 and Ω-6 polyunsaturated fatty acids, and sulfur-containing compounds, can influence gene expression through *epigenetic mechanisms*. These compounds may regulate the production of proinflammatory proteins and modulate immune responses in MS [63].

### 5.1. Vitamin D3

Vitamin D3, also known as cholecalciferol, is a form of vitamin D that is synthesized in the skin when exposed to sunlight or obtained through dietary sources. It plays a crucial role in maintaining calcium and phosphate balance in the body, which is essential for bone health. However, emerging research suggests that vitamin D3 also has important roles in brain plasticity and MS [64].

Vitamin D3 has been found to have immunomodulatory and neuroprotective effects within the central nervous system. It acts through its receptors in glial cells, such as astrocytes and microglia, to influence the production of proinflammatory cytokines and antioxidants. This modulation of neuroinflammation may help mitigate the cascade of events leading to neuronal damage. Vitamin D3 also contributes to neurogenesis and synaptic plasticity, which are important processes for brain plasticity [65].

In the context of MS, vitamin D3 has been investigated for its potential therapeutic effects. Studies have shown that vitamin D3 supplementation may reduce disease activity and demyelination in animal models of MS. Clinical studies have also suggested that vitamin D3 deficiency is associated with an increased risk of developing MS and that supplementation may have beneficial effects on disease progression and activity [66].

In a study on mice with experimental autoimmune encephalomyelitis (EAE), an animal model of MS, early intervention with the active form of vitamin D3 (1,25-dihydroxyvitamin D3) was found to control neuroinflammation and reduce inflammation, demyelination, and oxidative stress. This suggests that vitamin D3 may have potential as an adjunct therapy for the MS population [64].

Low vitamin D during childhood may increase the risk of developing multiple sclerosis (MS) later in life, as neuron-specific vitamin D signaling helps promote an anti-inflammatory state in microglia, offering protection against central nervous system autoimmunity [67].

Vitamin D3’s role in modulating neuroinflammation and its potential neuroprotective effects make it an interesting target for further research and therapeutic strategies in neurodegenerative and neuropsychiatric disorders. However, more clinical studies are needed to validate these findings and fully understand the mechanisms and benefits of vitamin D3 supplementation in these conditions.

In summary, vitamin D3 plays a role in brain plasticity and has been investigated for its potential therapeutic effects in multiple sclerosis. It has immunomodulatory and neuroprotective properties that may help reduce neuroinflammation and promote neurogenesis and synaptic plasticity. However, further research is needed to fully understand the benefits and mechanisms of vitamin D3 in brain health and disease.

### 5.2. Immunoglobulin Y

Immunoglobulin Y (IgY) is a type of immunoglobulin, or antibody, found in the blood of birds, reptiles, and amphibians. It is the avian equivalent of mammalian immunoglobulin G (IgG) [68]. In these animals, IgY is produced by specialized cells, called B lymphocytes, and plays a crucial role in their immune defense against pathogens. One unique characteristic of IgY is that it is actively transported from the bloodstream into the egg yolk during egg formation [68]. As a result, IgY is present in copious quantities in the egg yolk, providing passive immunity to the developing embryo or offspring. This is an evolutionary adaptation that allows birds and reptiles to pass on immune protection to their young before they have fully developed their own immune systems [68]. IgY has gained attention in biomedical research and applications due to its potential as an alternative to mammalian antibodies, such as IgG [68]. It offers several advantages, including ethical considerations (no need for animal bleeding as antibodies are extracted from eggs), cost-effectiveness (production from egg yolks), and potential therapeutic applications [68]. Research on IgY has explored its use in various fields, including immunodiagnostics, immunotherapy, and antimicrobial applications. Studies have investigated its effectiveness in detecting and treating infectious diseases, as well as its potential in modulating immune responses and targeting specific pathogens [68,69].

There is limited research specifically investigating the role of IgY in MS. However, a study mentioned that IgY supplements derived from chicken egg yolk have shown potential in modulating gut microbiota and immune responses and have been effective against various infections [70]. In terms of MS, the study mentioned that clinical trials using IgY supplements in MS are limited but have shown positive outcomes, including reduced symptoms and altered immune responses [70]. However, further research is needed to understand the mechanisms of IgY’s interaction with gut microbiota, determine optimal dosage, and assess long-term safety [70].

### 5.3. Homeopathy and Alternative Medicine

Homeopathy is a form of complementary and alternative medicine (CAM) that is sometimes used by people with MS [11,12,71]. It is relatively cheap, and some find relief in it. While there is no definitive treatment for MS, management is largely focused on symptom relief [11,12]. Conventional therapies may not be effective for everyone or may cause unacceptable side effects [11,12,71,72]. As a result, some individuals with MS turn to CAM, including homeopathy, to help manage their symptoms. Homeopathy is a whole-person approach that allows for individualized treatment based on the specific symptoms and needs of the person with MS. It aims to stimulate the body’s self-healing mechanisms by administering highly diluted substances that would produce similar symptoms in a healthy person [11]. Homeopathic treatments for MS symptoms such as urinary incontinence, sexual dysfunction, cramps and spasms, tremors, and trigeminal neuralgia have shown some benefit in certain individuals [11,71,72,73]. However, it is important to note that the evidence supporting the effectiveness of homeopathy in treating MS is limited [11]. Studies on the use of homeopathy in MS have mainly focused on individual cases or small groups of populations, making it difficult to draw definitive conclusions [71,72,73]. Additionally, the placebo effect may play a role in the perceived benefits of homeopathic treatments [71,72,73].

Overall, while some individuals with MS may find relief from certain symptoms through homeopathy, more research is needed to determine its efficacy and safety in the management of MS [12,74].

### 5.4. Ginkgo biloba

*Ginkgo biloba* extract (GBE) is a plant extract derived from the leaves of the *Ginkgo biloba* tree. It has been used for centuries in traditional medicine and is now commonly consumed as a dietary supplement [75].

The presumed effects of GBE on brain plasticity are based on its potential neuroprotective and neuroregenerative properties. GBE has been shown to have antioxidant and anti-inflammatory effects, which can help protect neurons from damage and promote their survival [75]. It has also been found to enhance cerebral blood flow and improve vascular function, which is important for delivering oxygen and nutrients to the brain [75]. In terms of brain plasticity, GBE has been studied for its effects on synaptic plasticity, which is the ability of synapses to change and adapt in response to experience [75]. Some studies have suggested that *Ginkgo biloba* extract can enhance synaptic plasticity by modulating neurotransmitter pathways and promoting the release of endogenous relaxing factors [75]. These effects may contribute to improved cognitive function and memory.

There is limited evidence on the specific effects of GBE on brain plasticity in MS. However, some studies have investigated the effects of GBE on cognitive function and neuroprotection in MS.

One study evaluated the effects of GBE on cognitive function in individuals with MS and found that treatment with GBE did not improve cognitive performance [76]. Another study investigated the effects of GBE on cognitive performance in MS and found that GBE did not show a statistically significant improvement in cognitive function, except for a trend toward an improvement in cognitive testing [77].

While these studies focused on cognitive function, they did not specifically examine brain plasticity. However, GBE has been shown to have neuroprotective effects in various neurological diseases. It has been found to promote myelin generation, regulate the balance of microglia and astrocytes, and induce the generation of oligodendrocyte precursor cells, which are important for remyelination [78]. These effects may indirectly contribute to brain plasticity in MS.

Overall, the evidence on the effects of GBE on brain plasticity in MS is limited. Further research is needed to determine the specific effects of *Ginkgo biloba* on brain plasticity in individuals with MS.

### 5.5. Alpha Lipoic Acid

Alpha lipoic acid (ALA) is a natural compound with antioxidant and anti-inflammatory properties. It acts as a cofactor for mitochondrial enzymes involved in energy production and has various biological functions, including scavenging reactive oxygen species, regenerating other antioxidants, and modulating signal transduction pathways [79].

In terms of brain plasticity, ALA has been studied for its potential neuroprotective effects after brain injury. Animal studies have shown that ALA can stimulate the synthesis of glutathione, decrease cell death, promote angiogenesis, and reduce the formation of glial scars, which allows for the formation of new neural tissue [80]. These findings suggest that ALA may have a role in promoting brain repair and regeneration after injury.

Regarding MS, ALA has shown promise in preclinical and clinical studies. In animal models of MS, ALA has been found to reduce nerve damage, inhibit the migration of inflammatory cells, and decrease the activity of matrix metalloproteinases, which are involved in the breakdown of the blood–brain barrier [80]. Clinical trials have demonstrated the positive effects of ALA on cellular and physical outcomes in people with MS [81].

In summary, ALA has potential roles in brain plasticity by promoting neuroprotection and repair after injury. In the context of multiple sclerosis, ALA has shown promising effects in preclinical and clinical studies, but further research is needed to establish its efficacy as a therapy for MS.

### 5.6. Biotin

Biotin, also known as vitamin B7 or vitamin H, is a water-soluble vitamin that plays a crucial role in various metabolic processes in the body. It acts as a cofactor for several carboxylase enzymes involved in energy production, fatty acid synthesis, and amino acid metabolism.

In the context of MS, there is growing interest in the potential role of high-dose biotin in preventing brain neurodegeneration.

Several studies have investigated the effects of high-dose biotin (MD1003) in populations with progressive MS. These studies have shown promising results, suggesting that biotin may have a beneficial effect on disease progression and disability improvement [4,82,83,84]. The exact mechanisms by which biotin exerts its neuroprotective effects are not fully understood, but several hypotheses have been proposed.

1. One hypothesis is that biotin enhances energy production in demyelinated axons, which can help support their function and prevent neurodegeneration [60]. Biotin is involved in ATP production, which is essential for maintaining neuronal health and function [82].
2. Another hypothesis is that biotin promotes myelin synthesis in oligodendrocytes, the cells responsible for producing myelin, the protective covering of nerve fibers [60]. Biotin may enhance myelin production and repair, potentially slowing down disease progression [85].

It is important to note that while few studies have shown positive effects of high-dose biotin in MS, not all studies have reported significant benefits [85].

In summary, biotin is a vitamin that plays a role in various metabolic processes in the body. High-dose biotin has shown promise in preventing brain neurodegeneration in multiple sclerosis, potentially through its effects on energy production and myelin synthesis. However, more research is needed to confirm these findings and determine the optimal use of biotin in MS treatment.

### 5.7. Flavonoids

Flavonoids are a large group of plant polyphenols that are widely distributed in the human diet. They are found in various fruits, vegetables, nuts, and beverages [86].

Flavonoids have been studied for their potential health benefits and are believed to have several presumed effects on the human body. Flavonoids can reduce free radical formation and scavenge free radicals, which can help protect cells from oxidative damage [86]. They can also increase the plasma antioxidant status and preserve the levels of antioxidants like vitamin E [86]. They can modulate intracellular signaling cascades and inhibit enzymes involved in cellular activation, leading to a reduction in inflammation [87].

Flavonoids have been associated with a lower risk of cardiovascular diseases. They have been shown to have vasodilating actions, reduce lipid peroxidation, and protect against oxidative stress, which are all beneficial for heart health [88]. Flavonoids have immunomodulatory properties and can influence immune responses. Flavonoids have been studied for their pro-cognitive effects [86]. They can interact with major signal transduction cascades in the brain and impact the transcription factors involved in neuronal function [89].

There is limited direct evidence on the effects of flavonoids on brain plasticity, specifically in MS. However, flavonoids have been shown to have neuroprotective and neuroplasticity-promoting effects in various neurological conditions.

1. Flavonoids have been found to exert neuroprotective effects by reducing oxidative stress and inflammation, which are key factors in MS [90,91]. They have also been shown to promote synaptogenesis and neurogenesis, which are important processes for brain plasticity [90]. Additionally, flavonoids have been found to modulate signaling pathways involved in neuronal survival and synaptic plasticity [92,93].
2. In animal models of MS, flavonoids have demonstrated positive therapeutic effects. For example, flavonoid luteolin has been shown to suppress clinical symptoms, reduce inflammation, and prevent relapse in rats with EAE [94]. A systematic review of studies on EAE and MS also reported positive outcomes for the therapeutic effect of flavonoids on these conditions [95].

While the direct effects of flavonoids on brain plasticity in MS are not well studied, their neuroprotective and neuroplasticity-promoting properties suggest that they may have potential benefits for enhancing brain plasticity in MS. Further research is needed to explore the specific effects of flavonoids on brain plasticity in MS and to determine optimal dosages and treatment strategies [90,91,92,95].

### 5.8. Polyunsaturated Fatty Acids

Polyunsaturated fatty acids (PUFAs) are a type of fat that is essential for human health and cannot be produced by the body, so they must be obtained through diet. PUFAs can be further classified into two main groups: omega-3 (n-3) and omega-6 (n-6) fatty acids.

The presumed effects of PUFAs in humans are diverse and have been extensively studied. PUFAs, particularly omega-3 fatty acids, have been associated with a reduced risk of cardiovascular diseases. They can help lower blood triglyceride levels, reduce inflammation, improve blood vessel function, and prevent the formation of blood clots. Omega-3 fatty acids, especially docosahexaenoic acid (DHA), play a crucial role in brain development and function [96,97,98]. They are important for cognitive function, memory, and mood regulation. Adequate intake of omega-3 fatty acids has been linked to a lower risk of neurodegenerative diseases and improved mental health [96,97,98,99,100]. They also provide anti-inflammatory properties [97]. They can help reduce chronic inflammation in the body and modulate immune responses. This can be beneficial for conditions such as rheumatoid arthritis, inflammatory bowel disease, and asthma [97,99,101]. Some studies suggest that omega-3 fatty acids may have a protective effect against certain types of cancer, such as breast, colorectal, and prostate cancer [100,101,102]. However, more research is needed to fully understand the relationship between PUFAs and cancer prevention. PUFAs can influence metabolic processes, including lipid metabolism, insulin sensitivity, and glucose regulation. They may help improve insulin resistance, reduce the risk of type 2 diabetes, and prevent fatty liver disease [103].

It is important to note that the effects of PUFAs can vary depending on the specific type and ratio of omega-3 to omega-6 fatty acids consumed, as well as individual factors such as genetics, overall diet, and health status. Therefore, it is recommended to maintain a balanced intake of both omega-3 and omega-6 fatty acids for optimal health benefits [96,97,98,99,100,101,102,103].

The effects of PUFAs on brain plasticity in the MS population have been investigated in several studies. Their main effects can be concluded as follows:

1. A study on mice with induced CNS demyelination found that an increased n-3 PUFA status *promoted remyelination* after toxic injury to CNS oligodendrocytes. This effect may be mediated by n-3 PUFA-derived lipid metabolites [104].
2. Omega-3 PUFAs, such as docosahexaenoic acid (DHA) and eicosapentaenoic acid (EPA), have been shown to modulate microglial responses to myelin pathology. They can inhibit inflammation while *enhancing beneficial immune responses*, such as microglial phagocytosis. In a mouse model of MS, n-3 PUFA supplementation reduced demyelination and shifted microglial polarization toward a beneficial phenotype [105].
3. In vitro studies using oligodendroglia cells and primary oligodendrocytes have shown that supplementation with n-3 and n-6 PUFAs can promote *oligodendrocyte differentiation*. This was evidenced by an increased expression of markers of oligodendroglia differentiation and enhanced myelin sheet formation [105].
4. A study on healthy older adults found that omega-3 PUFAs were associated with individual differences in *functional brain connectivity*. Specifically, they were linked to connectivity within regions supporting executive function, memory, and emotion. These regions were also found to predict general, fluid, and crystallized intelligence [106].

### 5.9. Curcumin

Curcumin is a natural compound found in turmeric, which is a spice commonly used in cooking. It has been extensively studied for its potential health benefits [107]. Curcumin is known for its antioxidant, anti-inflammatory, and anticancer properties. It has been shown to have diverse pharmacological effects, including neuroprotective, cardioprotective, and immunomodulatory activities [107,108]. Curcumin has been investigated for its potential in the prevention and treatment of various diseases, such as cancer, cardiovascular diseases, diabetes, arthritis, neurological diseases, and gastrointestinal disorders [107,108,109,110,111,112]. However, curcumin’s low bioavailability has been a challenge in harnessing its full therapeutic potential [107,108,109,110,111,112].

There is limited research specifically investigating the effect of curcumin on brain plasticity in MS populations. However, curcumin has been shown to have beneficial effects on brain health through various mechanisms, including its antioxidant, anti-inflammatory, and neuroprotective properties. These effects may potentially contribute to the modulation of brain plasticity in MS populations.

Curcumin has been studied in animal models of MS, where it has demonstrated neuroprotective effects, reduced demyelination, and improved cognitive function. Further research is needed to determine the specific impact of curcumin on brain plasticity in MS human populations [113,114,115].

### 5.10. Resveratrol

Resveratrol is a natural compound found in various plants, including grapes, berries, peanuts, and red wine [116,117]. It is a polyphenol with antioxidant, anti-inflammatory, and anticancer properties. Resveratrol has been studied for its potential health benefits in several areas [116]. Resveratrol may have cardioprotective effects by improving inflammatory markers, atherogenic profile, glucose metabolism, and endothelial function [116,117]. It has shown promising results in preventing or delaying the development of various types of cancer, including prostate cancer by inhibiting proliferation, promoting apoptosis, and enhancing sensitivity to radiation [118]. Also, it has been investigated for its potential in neurodegenerative diseases such as Alzheimer’s disease (AD), Parkinson’s disease (PD), and Huntington’s disease (HD).

It has shown effects in in vitro models, but further research is needed [119]. Resveratrol has been associated with improved insulin sensitivity, increased energy expenditure, and a decrease in body fat [120]. It may also have antiobesity effects by inhibiting adipogenesis and increasing lipid mobilization [121]. Also, it has been shown to reduce inflammation and oxidative stress in various tissues and organs [117]. It may also have antiangiogenic effects [118].

It is important to note that while resveratrol has shown promising results in preclinical studies and some clinical trials, there is still a need for more research, especially in human subjects, to fully understand its effects and determine optimal doses [122,123]. Additionally, the bioavailability of resveratrol and its potential interactions with medications should be considered [121].

There is limited research specifically investigating the effects of resveratrol on brain plasticity in MS populations. In an EAE mouse model of MS, resveratrol treatment was found to improve clinical outcomes and protect against optic nerve and spinal cord degeneration [124]. Another study in cuprizone-intoxicated mice, a model of demyelination/remyelination like MS, showed that resveratrol enhanced motor coordination, reversed demyelination, and increased the expression of genes associated with active remyelination [125].

While these studies suggest potential neuroprotective effects of resveratrol in MS models, it is important to note that the effects of resveratrol on brain plasticity in human MS populations have not been extensively studied. Further research is needed to determine the specific effects of resveratrol on brain plasticity in MS populations and its potential as a therapeutic intervention for MS-related neurodegenerative processes [124].

### 5.11. Terpenoids

Terpenoids are a class of naturally occurring compounds that are derived from terpenes. They are found in various plants, animals, and microorganisms. Terpenoids have diverse biological activities and are known for their pharmacological effects on the human body.

Terpenoids have been studied for their potential therapeutic benefits in various chronic illnesses. They have been found to have anti-inflammatory, antimicrobial, antifungal, antiviral, antitumor, and antioxidant properties [126,127,128,129,130,131,132,133]. They can also act as skin penetration enhancers and have been used in the prevention and treatment of inflammatory diseases [128]. In the context of specific diseases, terpenoids have shown promise in the treatment of non-alcoholic fatty liver disease. They regulate lipid metabolism disorder, insulin resistance, oxidative stress, and inflammation, and target pathways such as AMPK, PPARs, Nrf-2, and SIRT 1 [126]. Additionally, terpenoids have been explored for their potential antiviral efficacy against SARS-CoV-2, the virus responsible for COVID-19 [15].

Furthermore, terpenoids have been investigated for their effects on psychiatric disorders. Preclinical studies have demonstrated their neuropharmacological effects and their potential as alternative therapeutic options for psychiatric disorders [130].

Two studies provide insights into the effects of terpenoids on brain plasticity in MS populations. In a study by Tomassini et al., it was found that inflammation in MS interferes with brain plasticity [134]. However, a pharmacological reduction in inflammation, such as with interferon beta treatment, can restore brain plasticity. The study used a visuomotor adaptation task and functional MRI to assess brain plasticity in MS populations. The results showed that reduced inflammation with IFN beta treatment led to the restoration of brain plasticity, suggesting that the modulation of inflammation can enhance recovery-oriented strategies that rely on brain plasticity. Similarly, Shin et al. investigated the effects of terpenes on neuronal health in Drosophila AD models. They found that certain terpenes, including limonene, had neuroprotective effects against the neurotoxicity of beta-amyloid 42 (Aβ42), a protein associated with AD. Limonene treatment decreased cell death, inflammation, and oxidative stress in the brains of the AD model flies. While this study was not conducted specifically in MS populations, it provides insights into the potential neuroprotective effects of terpenes on brain health [129].

These findings suggest that terpenoids, including terpenes like limonene, may have the potential to modulate brain plasticity in MS populations by reducing inflammation, promoting neuroprotection, and enhancing recovery processes.

### 5.12. Polyphenols

Polyphenols are a group of natural compounds found in a variety of plant-based foods, such as fruits, vegetables, nuts, seeds, whole grains, tea, coffee, and red wine. They are characterized by the presence of phenol structural units and are known for their antioxidant properties. The presumed effects of polyphenols in the human body are diverse and have been the subject of extensive research. Some of their potential benefits include the following:

1. Polyphenols can scavenge and neutralize harmful free radicals, *reducing oxidative stress* and protecting cells from damage [135].
2. They carry potential *lipid-lowering and atheroprotective effects*. They may help reduce the oxidation of LDL cholesterol, improve endothelial function, lower blood pressure, and inhibit platelet aggregation [135,136].
3. Polyphenols possess *anti-inflammatory properties* and can help modulate the immune system by affecting the production of cytokines and other factors involved in the immune response [137,138].
4. Polyphenols have been investigated for their potential *anticarcinogenic effects*. They may inhibit tumor growth, induce apoptosis in cancer cells, and have antimutagenic properties [139].
5. Some polyphenols, such as resveratrol and epigallocatechin-3-gallate, have shown promise in protecting against neurodegenerative disorders by *reducing mitochondrial dysfunction and oxidative stress* [140].

There is evidence that the use of plant polyphenols may have beneficial effects on brain plasticity in MS. For example, grape seed extract has been shown to have neuroprotective properties and can improve brain plasticity in an EAE mouse model of MS. GSE treatment in EAE mice resulted in the correction of oxidative stress damage, the restoration of antioxidant capacities, the normalization of myelin protein expression, and the modulation of astroglial and microglial proliferation. These effects suggest that GSE may have a positive impact on brain plasticity in MS [141].

### 5.13. Sulfur-Containing Compounds

Sulfur-containing compounds play important roles in the human body and have various effects. Methionine, cysteine, homocysteine, and taurine are sulfur-containing amino acids. They are essential for protein synthesis and are involved in antioxidant defense mechanisms. They contribute to the synthesis of intracellular antioxidants like glutathione, which helps protect cells from oxidative damage [142].

Glutathione and N-acetylcysteine are derivatives of sulfur-containing amino acids and act as powerful antioxidants. They help neutralize free radicals and protect cells from oxidative stress. They are also used in chelation therapy to eliminate toxic metals from the body [142]. Naturally occurring sulfur-containing ligands can act as detoxifying agents, preventing the toxic effects of heavy and transition metal ions and aiding in their elimination from the body [142]. Exposure to malodorous sulfur compounds has been associated with respiratory symptoms such as cough, respiratory infections, and nasal symptoms. A reduction in sulfur compound exposure has been shown to decrease the frequency of these symptoms [143,144]. Some sulfur compounds, such as diallyl sulfide and diallyl disulfide, have immunomodulatory effects. They can enhance white blood cell count, antibody production, and bone marrow cellularity, suggesting potential immunostimulant effects [145]. Sulfur-containing antioxidants, such as N-acetylcysteine and lipoic acid, have been shown to reduce oxidative stress induced by substances like lead. They can help protect against oxidative damage and maintain cellular health [146].

One study investigated the therapeutic effects of hydrogen sulfide (H2S) in a cuprizone-induced MS model in mice. The study found that treatment with a hydrogen sulfide donor improved locomotor coordination, reduced neuronal inflammation and demyelination, and decreased oxidative stress in the brain. These effects were associated with the downregulation of miR-146a expression and the regulation of the miR-146a/NF-κB/IL-1β axis [147].

Another study examined the effects of sulfur dioxide exposure on synaptic plasticity in the hippocampus of rats. The study found that exposure to SO_2_ at different concentrations and durations resulted in the inhibition of synaptic plasticity markers and memory-related proteins in the hippocampus. The effects of SO_2_ on synaptic plasticity were dependent on the duration and concentration of exposure [148].

Several dietary nutraceuticals have been tested in MS; while some have the potential to aid the process of neuroregeneration, some still need research. A summary of the key current knowledge is found in Table 2.

## 6. Obesity

Obesity is considered both a risk for MS development and the progression of the disease [151,152]. Obesity is a medical condition characterized by excessive accumulation of body fat, which can have negative effects on health. Obesity is defined using the body mass index (BMI), which is calculated by dividing a person’s weight in kilograms by the square of their height in meters. A BMI of 30 or higher equals obesity. The prevalence of obesity varies across different populations and countries. In developed countries, such as the United States, Europe, and Australia, the average prevalence of obesity is estimated to be around 15–20% [153]. However, there are significant variations within and between countries.

A study conducted in the Nurses’ Health Study and Nurses’ Health Study II found that obesity at age 18 was associated with a more than twofold increased risk of MS [151]. Another study using Mendelian randomization analysis found that genetic BMI was associated with a 41% increased risk of MS [154]. Furthermore, a meta-analysis of observational studies reported that excess body weight during childhood and adolescence increased the risk of MS, particularly in females [155]. Another study found that obese adolescents had a higher risk of developing MS compared to non-obese individuals, even after adjusting for other factors [156,157]. Another study found that a common polymorphism in the fat-mass obesity gene, associated with obesity, was linked to being overweight/obese in MS populations [158].

In terms of disease progression, a study showed that overweight or obese MS populations had higher MRI activity and were less likely to achieve no evidence of disease activity status compared to normal-weight populations [159]. Additionally, obesity was associated with a higher risk of disability accumulation in MS populations [160]. The mechanisms underlying the association between obesity and MS that were suggested are chronic inflammation, altered gut microbiota, and vitamin D deficiency [161,162].

In conclusion, obesity, particularly during adolescence and early adulthood, is associated with an increased risk of developing MS and may also impact disease progression and disability accumulation. Obesity prevention in adolescence may reduce the risk of developing MS [156,157,163,164].

Obesity has been found to have an impact on brain plasticity. A study conducted on adult volunteers with a wide range of BMI found that the effect of short-term monocular deprivation, a measure of early visual plasticity, decreased with increasing BMI. Morbidly obese individuals also showed altered binocular rivalry dynamics compared to normal-weight individuals, indicating impaired sensory processing and plasticity [165,166].

Another study investigated the plasticity of the motor cortex in obese individuals compared to those with a healthy weight. It found that obese individuals had an impaired capacity for plasticity in the motor cortex, as measured by the suppression of cortical excitability following brain stimulation. This suggests that the ability of the motor cortex to change and adapt is reduced in obesity [167].

Furthermore, obesity has been associated with alterations in synaptic plasticity in the hippocampus, a brain region involved in learning and memory. Studies have shown that obese individuals, particularly males, exhibit deficits in hippocampal synaptic plasticity, which may contribute to cognitive impairments [168].

The underlying mechanisms linking obesity and impaired brain plasticity are not fully understood. However, factors such as chronic inflammation, oxidative stress, altered gut–brain hormonal functionality, and insulin resistance have been proposed as potential contributors [168,169].

**Figure 4:**
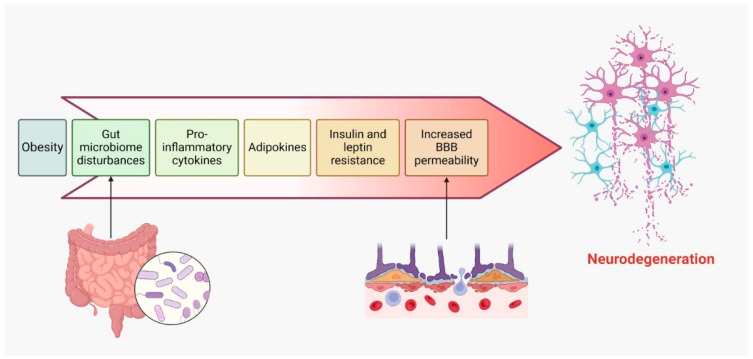
The cascade of mechanisms in obesity-induced neurodegeneration.

Obese populations with MS may experience neurodegeneration through several mechanisms, as shown in Figure 4.

1. Obesity is linked to a chronic, low-grade inflammatory state, marked by elevated levels of proinflammatory cytokines. This persistent systemic inflammation may extend to the central nervous system (CNS), where it exacerbates neuroinflammation and accelerates neurodegeneration in individuals with MS [170,171].
2. Adipose tissue in obese individuals secretes various adipokines, such as leptin, resistin, and visfatin, which are dysregulated in obesity. These adipokines can traverse the blood–brain barrier and activate immune cells within the CNS, thereby fostering inflammation and contributing to demyelination in MS [4,172].
3. Obesity can compromise the integrity of the blood–brain barrier (BBB), increasing its permeability and facilitating the entry of immune cells and inflammatory mediators into the CNS. This dysfunction of the BBB further drives neuroinflammation and neurodegeneration in MS patients [170,173].
4. High-calorie diets and obesity are often associated with disruptions in gut microbiota. Emerging research suggests that gut dysbiosis may influence the onset and progression of MS, as altered microbiota can affect immune responses and neuroinflammation, thereby worsening neurodegeneration in obese individuals with MS [162,174].
5. Obesity is commonly accompanied by insulin and leptin resistance, impairing neuroprotective signaling pathways. Resistance to these molecules may diminish their protective roles in the CNS, leading to increased neurodegeneration in MS patients [175].

Combating obesity is challenging for all populations, particularly those with MS. Regular exercise is essential for weight management, with the American College of Sports Medicine recommending 150–250 min of moderate-intensity activity weekly to prevent weight gain, and 225–420 min for weight loss in people with MS [176]. Exercise programs should be tailored to the specific needs and limitations of individuals with MS, aiming to exceed energy expenditure recommendations [176]. Behavioral weight loss interventions have demonstrated efficacy in achieving significant weight loss, enhancing mobility and quality of life, and reducing fatigue [177,178]. Bariatric surgery may be considered for severely obese individuals with MS, as it has proven safe and effective for weight loss and improved mobility [179,180]. While evidence for intermittent fasting in MS is limited, it may offer benefits similar to those seen in obesity and type 2 diabetes [180]. Tailored dietary strategies and nutrition education can significantly aid in managing metabolic comorbidities and supporting sustainable dietary changes [180].

Obesity during adolescence and early adulthood increases the risk of MS and worsens disease progression. Mechanisms include chronic inflammation, altered gut microbiota, and vitamin D deficiency, while obesity impairs brain plasticity and exacerbates neurodegeneration. Management strategies such as exercise, weight loss interventions, dietary changes, and intermittent fasting can help reduce obesity’s impact on MS [161,162,163,164,165,166,167,168,169,170,171,172,173,174,175,176,177,178,179,180].

## 7. Conclusions

MS presents a complex interplay of immune-mediated pathology, characterized by inflammation, demyelination, and neurodegeneration. The CNS undergoes significant changes due to the disease’s progression, leading to varied symptoms and disability levels among populations [1,2,3,4,5,6,7]. Enhancing brain plasticity offers a promising avenue for mitigating these effects and improving population outcomes [21,22,23].

Brain plasticity, the capacity of the brain to reorganize its structure and function, is vital for learning, memory, and recovery from injuries. In the context of MS, promoting brain plasticity can help counteract the neurological damage caused by the disease. Several mechanisms underpin brain plasticity, including synaptic modification, neurogenesis, and the activation of complex signaling pathways that support neuronal connectivity and survival [26,27,28].

Physical exercise is a potent modulator of brain plasticity. Aerobic exercise, resistance training, and even simple activities like walking have been shown to enhance synaptic plasticity, neurogenesis, and cognitive functions [29,30]. The increased production of BDNF is one key mechanism through which physical activity exerts its beneficial effects. BDNF supports the survival of existing neurons and encourages the growth and differentiation of new neurons and synapses [31,32].

Diet plays a crucial role in modulating brain health. Caloric restriction and intermittent fasting have been associated with an increased expression of neurotrophic factors and enhanced synaptic function [176,177,178,179,180]. Additionally, specific nutrients, such as omega-3 fatty acids found in fish oil, have anti-inflammatory properties that can help reduce CNS inflammation and promote brain plasticity [96,97,98,99,100,101,102,103,104,105,106,107,150]. Polyphenols, present in foods like dark chocolate and certain fruits, also contribute to neuroprotection and cognitive enhancement [116,117,118].

Obesity is a known risk factor for MS progression. Controlling body weight through diet and exercise can reduce inflammation and oxidative stress, thereby supporting brain plasticity. Nutraceuticals like ALA and resveratrol have been shown to reduce oxidative damage and support metabolic homeostasis, further aiding in the control of obesity-related impacts on brain health [151,152,153,154,155,156,157,158,159,160,161,162,163,164,165,166,167,168,169,170,171,172,173,174,175,176,177,178,179,180].

Nutraceuticals are bioactive compounds that provide health benefits beyond basic nutrition. They play a significant role in supporting brain plasticity and overall brain health in MS populations.

Vitamin D3 has immunomodulatory and neuroprotective effects. It influences the production of proinflammatory cytokines and antioxidants, aiding in the reduction in neuroinflammation and promoting neurogenesis and synaptic plasticity. Studies have shown that vitamin D3 supplementation can reduce disease activity and progression in MS [65,66,67].

Omega-3 fatty acids, particularly DHA, have been shown to support brain plasticity by modulating synaptic function and promoting neurogenesis. They also have anti-inflammatory properties that can help mitigate CNS inflammation in MS populations [96,97,98,99,100,101,102,103,104,105,106,150].

Antioxidants like ALA and flavonoids found in green tea and dark chocolate can scavenge free radicals, reducing oxidative stress and supporting neuroprotection. These compounds have shown potential in improving clinical outcomes in MS, including reducing fatigue and supporting cognitive functions [79,80,81].

GBE has been reported to improve cognitive functions and reduce fatigue in MS populations. Its antioxidant capacity supports brain plasticity by protecting neurons from oxidative damage and enhancing synaptic function [75,76,77,78].

Integrating lifestyle changes and nutraceuticals into the management of MS provides a holistic approach that complements traditional pharmacological treatments. By addressing modifiable risk factors and supporting brain plasticity, populations can experience improved neurological outcomes and quality of life. Regular physical activity, a balanced diet rich in neuroprotective nutrients, and the strategic use of nutraceuticals can form a comprehensive strategy for managing MS [171,172,173,174,175,176,177,178,179,180].

Future research should focus on the long-term effects of these lifestyle modifications and nutraceuticals on brain plasticity and MS progression. Large-scale, randomized controlled trials are needed to establish definitive evidence and provide clear guidelines for clinicians. Understanding the individual variability in response to these interventions can also help tailor personalized treatment plans for MS populations.

In conclusion, the synergistic effects of lifestyle changes and nutraceuticals hold great promise for enhancing brain plasticity and improving the lives of those living with MS. By adopting these strategies, populations can take an active role in managing their condition and optimizing their neurological health.

## Figures and Tables

**Table 1:** Modifiable risk and protective factors of neurodegeneration in MS.

Risk Factors	Protective Factors
*Smoking*	Smoking cessation
*Obesity*	Calorie restriction Physical activity
*Childhood low sun exposure*	Higher sun exposure Vitamin D3 supplementation
*Oxidative diet*	Antioxidant supplementation Diet re-assessment

**Table 2:** Summary of nutraceuticals used in MS.

Compound	Primary Function/Effect	Role in Brain Plasticity and MS	Key Findings and Studies
***Vitamin D3 (Cholecalciferol)***	Maintains calcium and phosphate balance for bone health, with emerging roles in brain health.	Immunomodulatory and neuroprotective effects; promotes neurogenesis and synaptic plasticity.	May reduce disease activity and demyelination in MS; early intervention in EAE mice controlled neuroinflammation [64,65,66].
***Immunoglobulin Y (IgY)***	Avian antibody involved in immune defense; passive immunity to offspring via egg yolk.	Limited research in MS, but potential in modulating immune responses and gut microbiota.	Positive outcomes in clinical trials for MS, with reduced symptoms and altered immune responses; further research needed [68,69,70].
***Homeopathy***	CAM approach using highly diluted substances for symptom relief based on individualized treatment.	Limited evidence; potential placebo effect.	Some individuals report symptom relief; more research is required to determine efficacy in MS [12,71,72,73,74].
***Ginkgo biloba extract (GBE)***	Plant extract with neuroprotective and neuroregenerative properties; enhances cerebral blood flow.	Limited evidence in MS; potential effects on synaptic plasticity.	No significant cognitive improvement in MS; potential neuroprotective effects in neurological diseases [75,76,77,78].
***Alpha Lipoic Acid (ALA)***	Antioxidant and anti-inflammatory compound with roles in mitochondrial energy production.	Promotes neuroprotection and repair after injury; potential therapy for MS.	Preclinical and clinical studies show promising effects; more research needed to establish efficacy in MS [79,80,81].
***Biotin (Vitamin B7)***	Cofactor in metabolic processes, energy production, and fatty acid synthesis.	Potential in preventing brain neurodegeneration in MS.	High-dose biotin (MD1003) shows promise in slowing MS progression; mechanisms include enhanced energy production and myelin synthesis [4,60,82,83,84,85].
***Flavonoids***	Plant polyphenols with antioxidant, anti-inflammatory, and immunomodulatory properties.	Neuroprotective and neuroplasticity-promoting effects; limited direct evidence in MS.	Positive therapeutic effects in EAE models; potential benefits for brain plasticity in MS, but more research needed [86,87,88,89,90,91,92,93,94,95,149].
***Polyunsaturated Fatty Acids (PUFA)***	Essential fatty acids with anti-inflammatory properties and roles in brain function.	Modulate microglial responses; promote remyelination and oligodendrocyte differentiation.	Omega-3 PUFA supplementation reduces demyelination in MS models; associated with improved brain connectivity in humans [96,97,98,99,100,101,102,103,104,105,106,150].
***Curcumin***	Natural compound with antioxidant, anti-inflammatory, and neuroprotective properties.	Potential benefits for brain health and plasticity in MS, though limited research specific to MS.	Demonstrated neuroprotective effects and reduced demyelination in MS animal models; more research needed [107,108,109,110,111,112,113,114,115].
***Resveratrol***	Polyphenol with antioxidant, anti-inflammatory, and anti-cancer properties.	Limited research in MS; potential neuroprotective effects.	Improved outcomes in EAE mouse models; promotes remyelination and motor coordination in MS-like conditions in mice [116,117,118,119,120,121,122,123,124,125].
***Terpenoids***	Natural compounds with diverse biological activities, including anti-inflammatory and antioxidant effects.	Studied for potential benefits in chronic illnesses, including neurological conditions.	Preclinical studies suggest neuropharmacological effects; further research needed to explore benefits in MS and brain plasticity [126,127,128,129,130,131,132,133].

## Abbreviations

AD = Alzheimer’s disease; ALA = alpha lipoic acid; BDNF = brain-derived neurotrophic factor; CAM = complementary and alternative medicine; CIS = clinically isolated syndrome; CNS = central nervous system; CSF = cerebrospinal fluid; DHA = docosahexaenoic acid; DIS = dissemination in space; DIT = dissemination in time; EBV = Epstein–Barr virus; EAE = experimental autoimmune encephalomyelitis; EPA = eicosapentaenoic acid; GBE = *Ginkgo biloba* extract; HD = Huntington’s disease; MS = multiple sclerosis; OCBs = oligoclonal bands; PD = Parkinson’s disease; PPMS = primary progressive multiple sclerosis; PUFAs = polyunsaturated fatty acids; RRMS = relapsing–remitting multiple sclerosis; SPMS = secondary progressive multiple sclerosis; tPA = tissue-type plasminogen activator.

## References

[1] Ward M., Goldman M.D. (2022). Epidemiology and Pathophysiology of Multiple Sclerosis. Continuum.

[2] Dendrou C.A., Fugger L., Friese M.A. (2015). Immunopathology of multiple sclerosis. Nat. Rev. Immunol..

[3] Cree B.A., Gourraud P.A., Oksenberg J.R., Bevan C., Crabtree-Hartman E., Gelfand J.M., Goodin D.S., Graves J., Green A.J., University of California, San Francisco MS-EPIC Team (2016). Long-term evolution of multiple sclerosis disability in the treatment era. Ann. Neurol..

[4] Tarlinton R.E., Martynova E., Rizvanov A.A., Khaiboullina S., Verma S. (2020). Role of Viruses in the Pathogenesis of Multiple Sclerosis. Viruses.

[5] Vattoth S., Kadam G.H., Gaddikeri S. (2021). Revised McDonald Criteria, MAGNIMS Consensus and Other Relevant Guidelines for Diagnosis and Follow Up of MS: What Radiologists Need to Know?. Curr. Probl. Diagn. Radiol..

[6] Marrie R.A., Reider N., Cohen J., Stuve O., Sorensen P.S., Cutter G., Reingold S.C., Trojano M. (2014). A systematic review of the incidence and prevalence of autoimmune disease in multiple sclerosis. Mult. Scler. J..

[7] Nielsen N.M., Westergaard T., Frisch M., Rostgaard K., Wohlfahrt J., Koch-Henriksen N., Melbye M., Hjalgrim H. (2006). Type 1 diabetes and multiple sclerosis: A Danish population-based cohort study. Arch. Neurol..

[8] Barac I.S., Iancu M., Văcăraș V., Cozma A., Negrean V., Sâmpelean D., Mureșanu D.F., Procopciuc L.M. (2021). Potential Contribution of IL-27 and IL-23 Gene Polymorphisms to Multiple Sclerosis Susceptibility: An Association Analysis at Genotype and Haplotype Level. J. Clin. Med..

[9] Gupta G., Gelfand J.M., Lewis J.D. (2005). Increased risk for demyelinating diseases in populations with inflammatory bowel disease. Gastroenterology.

[10] Goris A., Vandebergh M., McCauley J.L., Saarela J., Cotsapas C. (2022). Genetics of multiple sclerosis: Lessons from polygenicity. Lancet Neurol..

[11] Vacaras V., Paraschiv A.-C., Iluț S., Vacaras C., Nistor C., Marin G.-E., Schiopu A.M., Nistor D.-T., Vesa Ș.C., Mureșanu D.F. (2024). Brain-Derived Neurotrophic Factor in Multiple Sclerosis Disability: A Prospective Study. Brain Sci..

[12] Cusick M.F., Libbey J.E., Fujinami R.S. (2013). Multiple sclerosis: Autoimmunity and viruses. Curr. Opin. Rheumatol..

[13] Taan M., Al Ahmad F., Ercksousi M.K., Hamza G. (2021). Risk Factors Associated with Multiple Sclerosis: A Case-Control Study in Damascus, Syria. Mult. Scler. Int..

[14] Jacobs B.M., Giovannoni G., Cuzick J., Dobson R. (2020). Systematic review and meta-analysis of the association between Epstein-Barr virus, multiple sclerosis and other risk factors. Mult. Scler..

[15] Xu Y., A Smith K., Hiyoshi A., Piehl F., Olsson T., Montgomery S. (2021). Hospital-diagnosed infections before age 20 and risk of a subsequent multiple sclerosis diagnosis. Brain.

[16] Jacobs B.M., Noyce A.J., Giovannoni G., Dobson R. (2020). BMI and low vitamin D are causal factors for multiple sclerosis: A Mendelian Randomization study. Neurol. Neuroimmunol. Neuroinflamm..

[17] Manouchehrinia A., Tench C.R., Maxted J., Bibani R.H., Britton J., Constantinescu C.S. (2013). Tobacco smoking and disability progression in multiple sclerosis: United Kingdom cohort study. Brain.

[18] Ramanujam R., Hedström A.-K., Manouchehrinia A., Alfredsson L., Olsson T., Bottai M., Hillert J. (2015). Effect of Smoking Cessation on Multiple Sclerosis Prognosis. JAMA Neurol..

[19] Sale A., Berardi N., Maffei L. (2014). Environment and brain plasticity: Towards an endogenous pharmacotherapy. Physiol. Rev..

[20] Chandler L. (2003). Ethanol and brain plasticity: Receptors and molecular networks of the postsynaptic density as targets of ethanol. Pharmacol. Ther..

[21] Kolb B., Whishaw I.Q. (1998). Brain plasticity and behavior. Annu. Rev. Psychol..

[22] Pascual-Leone A., Freitas C., Oberman L., Horvath J.C., Halko M., Eldaief M., Bashir S., Vernet M., Shafi M., Westover B. (2011). Characterizing brain cortical plasticity and network dynamics across the age-span in health and disease with TMS-EEG and TMS-fMRI. Brain Topogr..

[23] Missitzi J., Gentner R., Geladas N., Politis P., Karandreas N., Classen J., Klissouras V. (2011). Plasticity in human motor cortex is in part genetically determined. J. Physiol..

[24] Tzounopoulos T., Kraus N. (2009). Learning to encode timing: Mechanisms of plasticity in the auditory brainstem. Neuron.

[25] Travaglia A., Bisaz R., Cruz E., Alberini C.M. (2016). Developmental changes in plasticity, synaptic, glia and connectivity protein levels in rat dorsal hippocampus. Neurobiol. Learn. Mem..

[26] Johnston M.V. (2009). Plasticity in the developing brain: Implications for rehabilitation. Dev. Disabil. Res. Rev..

[27] Strettoi E., Di Marco B., Orsini N., Napoli D. (2022). Retinal Plasticity. Int. J. Mol. Sci..

[28] Pearson-Fuhrhop K.M., Kleim J.A., Cramer S.C. (2009). Brain plasticity and genetic factors. Top. Stroke Rehabil..

[29] Ding Q., Ying Z., Gómez-Pinilla F. (2011). Exercise influences hippocampal plasticity by modulating brain-derived neurotrophic factor processing. Neuroscience.

[30] Ben-Zeev T., Shoenfeld Y., Hoffman J.R. (2022). The Effect of Exercise on Neurogenesis in the Brain. Isr. Med. Assoc. J..

[31] Smith A.E., Goldsworthy M.R., Garside T., Wood F.M., Ridding M.C. (2014). The influence of a single bout of aerobic exercise on short-interval intracortical excitability. Exp. Brain Res..

[32] Dishman R.K., Berthoud H.R., Booth F.W., Cotman C.W., Edgerton V.R., Fleshner M.R., Gandevia S.C., Gomez-Pinilla F., Greenwood B.N., Hillman C.H. (2006). Neurobiology of exercise. Obesity.

[33] Achiron A., Kalron A. (2008). Physical activity: Positive impact on brain plasticity. Harefuah.

[34] Cirillo J., Lavender A.P., Ridding M.C., Semmler J.G. (2009). Motor cortex plasticity induced by paired associative stimulation is enhanced in physically active individuals. J. Physiol..

[35] Mori F., Kusayanagi H., Buttari F., Centini B., Monteleone F., Nicoletti C.G., Bernardi G., Di Cantogno E.V., Marciani M.G., Centonze D. (2013). Early treatment with high-dose interferon beta-1a reverses cognitive and cortical plasticity deficits in multiple sclerosis. Funct. Neurol..

[36] Parry A.M.M., Scott R.B., Palace J., Smith S., Matthews P.M. (2003). Potentially adaptive functional changes in cognitive processing for populations with multiple sclerosis and their acute modulation by rivastigmine. Brain.

[37] Murphy T., Dias G.P., Thuret S. (2014). Effects of diet on brain plasticity in animal and human studies: Mind the gap. Neural Plast..

[38] Gomez-Pinilla F., Ying Z. (2010). Differential effects of exercise and dietary docosahexaenoic acid on molecular systems associated with control of allostasis in the hypothalamus and hippocampus. Neuroscience.

[39] Xu B.-L., Wang R., Ma L.-N., Dong W., Zhao Z.-W., Zhang J.-S., Wang Y.-L., Zhang X. (2015). Effects of Caloric Intake on Learning and Memory Function in Juvenile C57BL/6J Mice. BioMed Res. Int..

[40] Kalantarzadeh E., Radahmadi M., Reisi P. (2022). The impact of different dark chocolate dietary patterns on synaptic potency and plasticity in the hippocampal CA1 area of the rats under chronic isolation stress. Nutr. Neurosci..

[41] Libner C.D., Salapa H.E., Levin M.C. (2020). The Potential Contribution of Dysfunctional RNA-Binding Proteins to the Pathogenesis of Neurodegeneration in Multiple Sclerosis and Relevant Models. Int. J. Mol. Sci..

[42] Dong Y., D’mello C., Pinsky W., Lozinski B.M., Kaushik D.K., Ghorbani S., Moezzi D., Brown D., Melo F.C., Zandee S. (2021). Oxidized phosphatidylcholines found in multiple sclerosis lesions mediate neurodegeneration and are neutralized by microglia. Nat. Neurosci..

[43] Gonsette R. (2008). Neurodegeneration in multiple sclerosis: The role of oxidative stress and excitotoxicity. J. Neurol. Sci..

[44] Vyshkina T., Kalman B. (2008). Autoantibodies and neurodegeneration in multiple sclerosis. Mod. Pathol..

[45] Kalman B., Leist T.P. (2003). A mitochondrial component of neurodegeneration in multiple sclerosis. Neuromol. Med..

[46] Mahad D.H., Trapp B.D., Lassmann H. (2015). Pathological mechanisms in progressive multiple sclerosis. Lancet Neurol..

[47] Barnett M.H., Mathey E., Kiernan M.C., Pollard J.D. (2016). Axonal damage in central and peripheral nervous system inflammatory demyelinating diseases: Common and divergent pathways of tissue damage. Curr. Opin. Neurol..

[48] Zoupi L., Booker S.A., Eigel D., Werner C., Kind P.C., Spires-Jones T.L., Newland B., Williams A.C. (2021). Selective vulnerability of inhibitory networks in multiple sclerosis. Acta Neuropathol..

[49] Chapman C., Lucas R.M., Ponsonby A.-L., Taylor B., Ausimmune Investigator Group (2022). Predictors of progression from a first demyelinating event to clinically definite multiple sclerosis. Brain Commun..

[50] Alroughani R.A., Akhtar S., Ahmed S.F., Al-Hashel J.Y. (2015). Clinical predictors of disease progression in multiple sclerosis populations with relapsing onset in a nation-wide cohort. Int. J. Neurosci..

[51] Simmons S.B., Schippling S., Giovannoni G., Ontaneda D. (2021). Predicting disability worsening in relapsing and progressive multiple sclerosis. Curr. Opin. Neurol..

[52] Motl R.W., Dlugonski D., Pilutti L., Sandroff B., McAuley E. (2012). Premorbid physical activity predicts disability progression in relapsing–remitting multiple sclerosis. J. Neurol. Sci..

[53] Lin X., Zarghami A., A Jelinek G., Simpson-Yap S., Neate S., Nag N. (2024). Diet and omega-3 and vitamin D supplement use predict five-year fatigue and disability trajectories in people with multiple sclerosis. Mult. Scler. Relat. Disord..

[54] Schmitz K., Barthelmes J., Stolz L., Beyer S., Diehl O., Tegeder I. (2015). “Disease modifying nutricals” for multiple sclerosis. Pharmacol. Ther..

[55] O’Connor K., Weinstock-Guttman B., Carl E., Kilanowski C., Zivadinov R., Ramanathan M. (2012). Patterns of dietary and herbal supplement use by multiple sclerosis populations. J. Neurol..

[56] Bergien S., Petersen C., Lynning M., Kristiansen M., Skovgaard L. (2020). Use of natural medicine and dietary supplements concomitant with conventional medicine among people with Multiple Sclerosis. Mult. Scler. Relat. Disord..

[57] Marx W., Hockey M., McGuinness A.J., Lane M., Christodoulou J., van der Mei I., Berk M., Dean O.M., Taylor B., Broadley S. (2019). The effect of emerging nutraceutical interventions for clinical and biological outcomes in multiple sclerosis: A systematic review. Mult. Scler. Relat. Disord..

[58] Spagnuolo P. (2015). Interactions Between Nutraceutical Supplements and Standard Acute Myeloid Leukemia Chemotherapeutics. J. Pharm. Pharm. Sci..

[59] Szymaszkiewicz A., López-Gómez L., Zielińska M., Abalo R. (2022). Nutraceuticals and peripheral glial cells: A possible link?. J. Integr. Neurosci..

[60] Yuan J., Tao Y., Wang M., Huang F., Wu X. (2024). Natural compounds as potential therapeutic candidates for multiple sclerosis: Emerging preclinical evidence. Phytomedicine.

[61] Rito Y., Torre-Villalvazo I., Flores J., Rivas V., Corona T. (2018). Epigenetics in Multiple Sclerosis: Molecular Mechanisms and Dietary Intervention. Central Nerv. Syst. Agents Med. Chem..

[62] Menéndez S.G., Manucha W. (2024). Vitamin D as a Modulator of Neuroinflammation: Implications for Brain Health. Curr. Pharm. Des..

[63] Wergeland S., Torkildsen Ø., Myhr K.-M., Aksnes L., Mørk S.J., Bø L. (2011). Dietary vitamin D3 supplements reduce demyelination in the cuprizone model. PLoS ONE.

[64] Soleimani M., Jameie S.B., Mehdizadeh M., Keradi M., Masoumipoor M., Mehrabi S. (2014). Vitamin D3 influence the Th1/Th2 ratio in C57BL/6 induced model of experimental autoimmune encephalomyelitis. Iran J. Basic Med. Sci..

[65] Dias da Silva W., Tambourgi D.V. (2010). IgY: A promising antibody for use in immunodiagnostic and in immunotherapy. Vet. Immunol. Immunopathol..

[66] Lee L., Samardzic K., Wallach M., Frumkin L.R., Mochly-Rosen D. (2021). Immunoglobulin Y for Potential Diagnostic and Therapeutic Applications in Infectious Diseases. Front. Immunol..

[67] Lee P.W., Selhorst A., Lampe S.G., Liu Y., Yang Y., Lovett-Racke A.E. (2020). Neuron-Specific Vitamin D Signaling Attenuates Microglia Activation and CNS Autoimmunity. Front. Neurol..

[68] Paraschiv A.C., Văcăraș V., Nistor C., Văcăraș C., Nistor D.T., Vesa Ș.C., Silvina I., Mureșanu D.F. (2024). Dysbiosis in Multiple Sclerosis: Can Immunoglobulin Y Supplements Help?. J. Gastrointestin Liver Dis..

[69] Shinto L., Calabrese C., Morris C., Sinsheimer S., Bourdette D. (2004). Complementary and alternative medicine in multiple sclerosis: Survey of licensed naturopaths. J. Altern. Complement. Med..

[70] Shinto L., Calabrese C., Morris C., Yadav V., Griffith D., Frank R., Oken B.S., Baldauf-Wagner S., Bourdette D. (2008). A Randomized pilot study of naturopathic medicine in multiple sclerosis. J. Altern. Complement. Med..

[71] Teixeira M.Z. (2013). Immunomodulatory drugs (natalizumab), worsening of multiple sclerosis, rebound effect and similitude. Homeopathy.

[72] Paraschiv A.-C., Vacaras V., Nistor C., Vacaras C., Strilciuc S., Muresanu D.F. (2024). The effect of multiple sclerosis therapy on gut microbiota dysbiosis: A longitudinal prospective study. Microb. Cell.

[73] Nguyen T., Alzahrani T. (2023). Ginkgo Biloba [Internet]. https://www.ncbi.nlm.nih.gov/books/NBK541024/.

[74] Lovera J.F., Kim E., Heriza E., Fitzpatrick M., Hunziker J., Turner A.P., Adams J., Stover T., Sangeorzan A., Sloan A. (2012). Ginkgo biloba does not improve cognitive function in MS: A randomized placebo-controlled trial. Neurology.

[75] Lovera J., Bagert B., Smoot K., Morris C.D., Frank R., Bogardus K., Wild K., Oken B., Whitham R., Bourdette D. (2007). Ginkgo biloba for the improvement of cognitive performance in multiple sclerosis: A randomized, placebo-controlled trial. Mult. Scler..

[76] Yin J.-J., He Y., An J., Miao Q., Sui R.-X., Wang Q., Yu J.-Z., Xiao B.-G., Ma C.-G. (2020). Dynamic Balance of Microglia and Astrocytes Involved in the Remyelinating Effect of Ginkgolide B. Front. Cell. Neurosci..

[77] Seifar F., Khalili M., Khaledyan H., Amiri Moghadam S., Izadi A., Azimi A., Shakouri S.K. (2019). α-Lipoic acid, functional fatty acid, as a novel therapeutic alternative for central nervous system diseases: A review. Nutr. Neurosci..

[78] Rocamonde B., Paradells S., Barcia J., Barcia C., Verdugo J.G., Miranda M., Gómez F.R., Soria J. (2012). Neuroprotection of lipoic acid treatment promotes angiogenesis and reduces the glial scar formation after brain injury. Neuroscience.

[79] Marracci G.H., McKeon G.P., Marquardt W.E., Winter R.W., Riscoe M.K., Bourdette D.N. (2004). Alpha lipoic acid inhibits human T-cell migration: Implications for multiple sclerosis. J. Neurosci. Res..

[80] Espiritu A.I., Remalante-Rayco P.P.M. (2021). High-dose biotin for multiple sclerosis: A systematic review and meta-analyses of randomized controlled trials. Mult. Scler. Relat. Disord..

[81] Levy M.J.F., Garcia-Diaz B., Sedel F., Evercooren A.B.-V., Mozafari S. (2022). High Dose Pharmaceutical Grade Biotin (MD1003) Accelerates Differentiation of Murine and Grafted Human Oligodendrocyte Progenitor Cells In Vivo. Int. J. Mol. Sci..

[82] Sedel F., Bernard D., Mock D.M., Tourbah A. (2016). Targeting demyelination and virtual hypoxia with high-dose biotin as a treatment for progressive multiple sclerosis. Neuropharmacology.

[83] Cree B.A.C., Cutter G., Wolinsky J.S., Freedman M.S., Comi G., Giovannoni G., Hartung H.P., Arnold D., Kuhle J., Block V. (2020). Safety and efficacy of MD1003 (high-dose biotin) in populations with progressive multiple sclerosis (SPI2): A randomised, double-blind, placebo-controlled, phase 3 trial. Lancet Neurol..

[84] Pietta P.G. (2000). Flavonoids as antioxidants. J. Nat. Prod..

[85] Ielpo M., Basile A., Miranda R., Moscatiello V., Nappo C., Sorbo S., Laghi E., Ricciardi M., Ricciardi L., Vuotto M. (2000). Immunopharmacological properties of flavonoids. Fitoterapia.

[86] Atucha N.M., Romecín P., Vargas F., García-Estañ J. (2022). Effects of Flavonoids in Experimental Models of Arterial Hypertension. Curr. Top. Med. Chem..

[87] Jaeger B.N., Parylak S.L., Gage F.H. (2017). Mechanisms of dietary flavonoid action in neuronal function and neuroinflammation. Mol. Asp. Med..

[88] Williamson G., Kay C.D., Crozier A. (2018). The Bioavailability, Transport, and Bioactivity of Dietary Flavonoids: A Review from a Historical Perspective. Compr. Rev. food Sci. food Saf..

[89] Cichon N., Saluk-Bijak J., Gorniak L., Przyslo L., Bijak M. (2020). Flavonoids as a Natural Enhancer of Neuroplasticity—An Overview of the Mechanism of Neurorestorative Action. Antioxidants.

[90] Vauzour D., Vafeiadou K., Rodriguez-Mateos A., Rendeiro C., Spencer J.P.E. (2008). The neuroprotective potential of flavonoids: A multiplicity of effects. Genes. Nutr..

[91] Bakoyiannis I., Daskalopoulou A., Pergialiotis V., Perrea D. (2019). Phytochemicals and cognitive health: Are flavonoids doing the trick?. Biomed. Pharmacother..

[92] Hendriks J.J.A., Alblas J., van der Pol S.M.A., van Tol E.A.F., Dijkstra C.D., de Vries H.E. (2004). Flavonoids influence monocytic GTPase activity and are protective in experimental allergic encephalitis. J. Exp. Med..

[93] Bayat P., Farshchi M., Yousefian M., Mahmoudi M., Yazdian-Robati R. (2021). Flavonoids, the compounds with anti-inflammatory and immunomodulatory properties, as promising tools in multiple sclerosis (MS) therapy: A systematic review of preclinical evidence. Int. Immunopharmacol..

[94] Marventano S., Kolacz P., Castellano S., Galvano F., Buscemi S., Mistretta A., Grosso G. (2015). A review of recent evidence in human studies of n-3 and n-6 PUFA intake on cardiovascular disease, cancer, and depressive disorders: Does the ratio really matter?. Int. J. Food Sci. Nutr..

[95] Chénais B., Blanckaert V. (2012). The janus face of lipids in human breast cancer: How polyunsaturated Fatty acids affect tumor cell hallmarks. Int. J. Breast Cancer.

[96] Davinelli S., Intrieri M., Corbi G., Scapagnini G. (2020). Metabolic indices of polyunsaturated fatty acids: Current evidence, research controversies, and clinical utility. Crit. Rev. Food Sci. Nutr..

[97] Colussi G., Catena C., Baroselli S., Nadalini E., Lapenna R., Chiuch A., Sechi L. (2007). Omega-3 fatty acids: From biochemistry to their clinical use in the prevention of cardiovascular disease. Recent. Patents Cardiovasc. Drug Discov..

[98] Khandelwal S., Kelly L., Malik R., Prabhakaran D., Reddy S. (2013). Impact of omega-6 fatty acids on cardiovascular outcomes: A review. J. Prev. Cardiol..

[99] D’archivio M., Scazzocchio B., Vari R., Santangelo C., Giovannini C., Masella R. (2018). Recent Evidence on the Role of Dietary PUFAs in Cancer Development and Prevention. Curr. Med. Chem..

[100] Mbarik M., Biam R.S., Robichaud P.-P., Surette M.E. (2020). The impact of PUFA on cell responses: Caution should be exercised when selecting PUFA concentrations in cell culture. Prostaglandins Leukot. Essent. Fat. Acids.

[101] Benatti P., Peluso G., Nicolai R., Calvani M. (2004). Polyunsaturated fatty acids: Biochemical, nutritional and epigenetic properties. J. Am. Coll. Nutr..

[102] Siegert E., Paul F., Rothe M., Weylandt K.H. (2017). The effect of omega-3 fatty acids on central nervous system remyelination in fat-1 mice. BMC Neurosci..

[103] Chen S., Zhang H., Pu H., Wang G., Li W., Leak R.K., Chen J., Liou A.K., Hu X. (2014). n-3 PUFA supplementation benefits microglial responses to myelin pathology. Sci. Rep..

[104] van Meeteren M., Baron W., Beermann C., Dijkstra C., van Tol E. (2006). Polyunsaturated fatty acid supplementation stimulates differentiation of oligodendroglia cells. Dev. Neurosci..

[105] Talukdar T., Zamroziewicz M.K., Zwilling C.E., Barbey A.K. (2018). Nutrient biomarkers shape individual differences in functional brain connectivity: Evidence from omega-3 PUFAs. Hum. Brain Mapp..

[106] Xu X.-Y., Meng X., Li S., Gan R.-Y., Li Y., Li H.-B. (2018). Bioactivity, Health Benefits, and Related Molecular Mechanisms of Curcumin: Current Progress, Challenges, and Perspectives. Nutrients.

[107] Tossetta G., Fantone S., Giannubilo S.R., Marzioni D. (2021). The Multifaced Actions of Curcumin in Pregnancy Outcome. Antioxidants.

[108] Ghafouri-Fard S., Shoorei H., Bahroudi Z., Hussen B.M., Talebi S.F., Taheri M., Ayatollahi S.A. (2022). Nrf2-Related Therapeutic Effects of Curcumin in Different Disorders. Biomolecules.

[109] Liu S., Liu J., He L., Liu L., Cheng B., Zhou F., Cao D., He Y. (2022). A Comprehensive Review on the Benefits and Problems of Curcumin with Respect to Human Health. Molecules.

[110] Abdollahi E., Momtazi A.A., Johnston T.P., Sahebkar A. (2018). Therapeutic effects of curcumin in inflammatory and immune-mediated diseases: A nature-made jack-of-all-trades?. J. Cell. Physiol..

[111] Sadek M.A., Rabie M.A., El Sayed N.S., Sayed H.M., Kandil E.A. (2023). Neuroprotective effect of curcumin against experimental autoimmune encephalomyelitis-induced cognitive and physical impairments in mice: An insight into the role of the AMPK/SIRT1 pathway. Inflammopharmacology.

[112] Elbini-Dhouib I., Manai M., Neili N.-E., Marzouki S., Sahraoui G., Ben Achour W., Zouaghi S., BenAhmed M., Doghri R., Srairi-Abid N. (2022). Dual Mechanism of Action of Curcumin in Experimental Models of Multiple Sclerosis. Int. J. Mol. Sci..

[113] Salehi B., Calina D., Docea A.O., Koirala N., Aryal S., Lombardo D., Pasqua L., Taheri Y., Castillo C.M.S., Martorell M. (2020). Curcumin’s Nanomedicine Formulations for Therapeutic Application in Neurological Diseases. J. Clin. Med..

[114] Kulashekar M., Stom S.M., Peuler J.D. (2018). Resveratrol’s Potential in the Adjunctive Management of Cardiovascular Disease, Obesity, Diabetes, Alzheimer Disease, and Cancer. J. Am. Osteopath. Assoc..

[115] Malhotra A., Bath S., Elbarbry F. (2015). An Organ System Approach to Explore the Antioxidative, Anti-Inflammatory, and Cytoprotective Actions of Resveratrol. Oxidative Med. Cell. Longev..

[116] Jasiński M., Jasińska L., Ogrodowczyk M. (2013). Resveratrol in prostate diseases—A short review. Central Eur. J. Urol..

[117] Rocha-González H.I., Ambriz-Tututi M., Granados-Soto V. (2008). Resveratrol: A natural compound with pharmacological potential in neurodegenerative diseases. CNS Neurosci. Ther..

[118] Springer M., Moco S. (2019). Resveratrol and Its Human Metabolites—Effects on Metabolic Health and Obesity. Nutrients.

[119] Berman A.Y., Motechin R.A., Wiesenfeld M.Y., Holz M.K. (2017). The therapeutic potential of resveratrol: A review of clinical trials. NPJ Precis. Oncol..

[120] Vang O., Ahmad N., Baile C.A., Baur J.A., Brown K., Csiszar A., Das D.K., Delmas D., Gottfried C., Lin H.-Y. (2011). What is new for an old molecule? Systematic review and recommendations on the use of resveratrol. PLoS ONE.

[121] Tomé-Carneiro J., Larrosa M., González-Sarrías A., Tomas-Barberan F.A., García-Conesa M.T., Espín J.C. (2013). Resveratrol and Clinical Trials: The Crossroad from In Vitro Studies to Human Evidence. Curr. Pharm. Des..

[122] Shamsher E., Khan R.S., Davis B.M., Dine K., Luong V., Somavarapu S., Cordeiro M.F., Shindler K.S. (2023). Nanoparticles Enhance Solubility and Neuroprotective Effects of Resveratrol in Demyelinating Disease. Neurotherapeutics.

[123] Ghaiad H.R., Nooh M.M., El-Sawalhi M.M., Shaheen A.A. (2016). Resveratrol Promotes Remyelination in Cuprizone Model of Multiple Sclerosis: Biochemical and Histological Study. Mol. Neurobiol..

[124] Yao P., Liu Y. (2022). Terpenoids: Natural Compounds for Non-Alcoholic Fatty Liver Disease (NAFLD) Therapy. Molecules.

[125] Ahmad A., Tiwari R.K., Ansari I.A. (2021). Revisiting the Antiviral Efficacy of Terpenoids: Plausible Adjunct Therapeutics for Novel SARS-CoV-2?. Endocr. Metab. Immune Disord. Drug Targets.

[126] Paduch R., Kandefer-Szerszeń M., Trytek M., Fiedurek J. (2007). Terpenes: Substances useful in human healthcare. Arch. Immunol. Ther. Exp..

[127] Shin M., Liu Q.F., Choi B., Shin C., Lee B., Yuan C., Song Y.J., Yun H.S., Lee I.-S., Koo B.-S. (2020). Neuroprotective Effects of Limonene (+) against Aβ42-Induced Neurotoxicity in a Drosophila Model of Alzheimer’s Disease. Biol. Pharm. Bull..

[128] Mony T.J., Elahi F., Choi J.W., Park S.J. (2022). Neuropharmacological Effects of Terpenoids on Preclinical Animal Models of Psychiatric Disorders: A Review. Antioxidants.

[129] Yang H., Dou Q.P. (2010). Targeting apoptosis pathway with natural terpenoids: Implications for treatment of breast and prostate cancer. Curr. Drug Targets.

[130] Heras B.d.L., Hortelano S. (2009). Molecular basis of the anti-inflammatory effects of terpenoids. Inflamm. Allergy-Drug Targets.

[131] Carsanba E., Pintado M., Oliveira C. (2021). Fermentation Strategies for Production of Pharmaceutical Terpenoids in Engineered Yeast. Pharmaceuticals.

[132] Tomassini V., D’Ambrosio A., Petsas N., Wise R.G., Sbardella E., Allen M., Tona F., Fanelli F., Foster C., Carnì M. (2016). The effect of inflammation and its reduction on brain plasticity in multiple sclerosis: MRI evidence. Hum. Brain Mapp..

[133] Cicero A.F., Colletti A. (2018). Polyphenols Effect on Circulating Lipids and Lipoproteins: From Biochemistry to Clinical Evidence. Curr. Pharm. Des..

[134] Giglio R.V., Patti A.M., Cicero A.F.G., Lippi G., Rizzo M., Toth P.P., Banach M. (2018). Polyphenols: Potential Use in the Prevention and Treatment of Cardiovascular Diseases. Curr. Pharm. Des..

[135] Gorzynik-Debicka M., Przychodzen P., Cappello F., Kuban-Jankowska A., Marino Gammazza A., Knap N., Wozniak M., Gorska-Ponikowska M. (2018). Potential Health Benefits of Olive Oil and Plant Polyphenols. Int. J. Mol. Sci..

[136] Focaccetti C., Izzi V., Benvenuto M., Fazi S., Ciuffa S., Giganti M.G., Potenza V., Manzari V., Modesti A., Bei R. (2019). Polyphenols as Immunomodulatory Compounds in the Tumor Microenvironment: Friends or Foes?. Int. J. Mol. Sci..

[137] Rodrigo R., Libuy M., Feliu F., Hasson D. (2014). Polyphenols in disease: From diet to supplements. Curr. Pharm. Biotechnol..

[138] Vacca R.A., Valenti D., Caccamese S., Daglia M., Braidy N., Nabavi S.M. (2016). Plant polyphenols as natural drugs for the management of Down syndrome and related disorders. Neurosci. Biobehav. Rev..

[139] Mabrouk M., El Ayed M., Démosthènes A., Aissouni Y., Aouani E., Daulhac-Terrail L., Mokni M., Bégou M. (2022). Antioxidant effect of grape seed extract corrects experimental autoimmune encephalomyelitis behavioral dysfunctions, demyelination, and glial activation. Front. Immunol..

[140] Colovic M.B., Vasic V.M., Djuric D.M., Krstić D.Z. (2018). Sulphur-Containing Amino Acids: Protective Role Against Free Radicals and Heavy Metals. Curr. Med. Chem..

[141] Partti-Pellinen K., Marttila O., Vilkka V., Jaakkola J.J.K., Jäppinen P., Haahtela T. (1996). The South Karelia Air Pollution Study: Effects of low-level exposure to malodorous sulfur compounds on symptoms. Arch. Environ. Health Int. J..

[142] Jaakkola J.J., Partti-Pellinen K., Marttila O., Miettinen P., Vilkka V., Haahtela T. (1999). The South Karelia Air Pollution Study: Changes in Respiratory Health in Relation to Emission Reduction of Malodorous Sulfur Compounds from Pulp Mills. Arch. Environ. Health Int. J..

[143] Gurvitz M., Lui G.K., Marelli A. (2020). Adult congenital heart disease—Preparing for the changing work force demand. Cardiol. Clin..

[144] Caylak E., Aytekin M., Halifeoglu I. (2008). Antioxidant effects of methionine, alpha-lipoic acid, N-acetylcysteine and homocysteine on lead-induced oxidative stress to erythrocytes in rats. Exp. Toxicol. Pathol..

[145] Ghaiad H.R., AAbd-Elmawla M., Gad E.S., A Ahmed K., Abdelmonem M. (2023). Modulating miR-146a Expression by Hydrogen Sulfide Ameliorates Motor Dysfunction and Axonal Demyelination in Cuprizone-Induced Multiple Sclerosis. ACS Chem. Neurosci..

[146] Yao G., Yun Y., Sang N. (2014). Differential effects between one week and four weeks exposure to same mass of SO_2_ on synaptic plasticity in rat hippocampus. Environ. Toxicol..

[147] Munger K.L., Chitnis T., Ascherio A. (2009). Body size and risk of MS in two cohorts of US women. Neurology.

[148] Neto A., Fernandes A., Barateiro A. (2023). The complex relationship between obesity and neurodegenerative diseases: An updated review. Front. Cell. Neurosci..

[149] Faysal M., Dehbia Z., Zehravi M., Sweilam S.H., Haque M.A., Kumar K.P., Chakole R.D., Shelke S.P., Sirikonda S., Nafady M.H. (2024). Flavonoids as Potential Therapeutics against Neurodegenerative Disorders: Unlocking the Prospects. Neurochem. Res..

[150] Lopresti A.L. (2022). Potential Role of Curcumin for the Treatment of Major Depressive Disorder. CNS Drugs.

[151] Seidell J.C. (2000). Obesity, insulin resistance and diabetes—A worldwide epidemic. Br. J. Nutr..

[152] Mokry L.E., Ross S., Timpson N.J., Sawcer S., Davey Smith G., Richards J.B. (2016). Obesity and Multiple Sclerosis: A Mendelian Randomization Study. PLoS Med..

[153] Liu Z., Zhang T.-T., Yu J., Liu Y.-L., Qi S.-F., Zhao J.-J., Liu D.-W., Tian Q.-B. (2016). Excess Body Weight during Childhood and Adolescence Is Associated with the Risk of Multiple Sclerosis: A Meta-Analysis. Neuroepidemiology.

[154] Hedström A.K., Olsson T., Alfredsson L. (2016). Body mass index during adolescence, rather than childhood, is critical in determining MS risk. Mult. Scler. J..

[155] Hedström A.K., Olsson T., Alfredsson L. (2012). High body mass index before age 20 is associated with increased risk for multiple sclerosis in both men and women. Mult. Scler. J..

[156] Kavak K.S., E Teter B., Hagemeier J., Zakalik K., Weinstock-Guttman B., on behalf of the New York State Multiple Sclerosis Consortium (2014). Higher weight in adolescence and young adulthood is associated with an earlier age at multiple sclerosis onset. Mult. Scler. J..

[157] Kvistad S.S., Myhr K.-M., Holmøy T., Benth J.Š., Wergeland S., Beiske A.G., Bjerve K.S., Hovdal H., Lilleås F., Midgard R. (2015). Body mass index influence interferon-beta treatment response in multiple sclerosis. J. Neuroimmunol..

[158] Lutfullin I., Eveslage M., Bittner S., Antony G., Flaskamp M., Luessi F., Salmen A., Gisevius B., Klotz L., Korsukewitz C. (2023). Association of obesity with disease outcome in multiple sclerosis. J. Neurol. Neurosurg. Psychiatry.

[159] Harroud A., Manousaki D., Butler-Laporte G., Mitchell R.E., Davey Smith G., Richards J.B., Baranzini S.E. (2021). The relative contributions of obesity, vitamin D, leptin, and adiponectin to multiple sclerosis risk: A Mendelian randomization mediation analysis. Mult. Scler. J..

[160] Samara A., Cantoni C., Piccio L., Cross A.H., Chahin S. (2023). Obesity, gut microbiota, and multiple sclerosis: Unraveling the connection. Mult. Scler. Relat. Disord..

[161] Hedström A.K., Bomfim I.L., Barcellos L., Gianfrancesco M., Schaefer C., Kockum I., Olsson T., Alfredsson L. (2014). Interaction between adolescent obesity and HLA risk genes in the etiology of multiple sclerosis. Neurology.

[162] Pakpoor J., Schmierer K., Cuzick J., Giovannoni G., Dobson R. (2020). Estimated and projected burden of multiple sclerosis attributable to smoking and childhood and adolescent high body-mass index: A comparative risk assessment. Leuk. Res..

[163] Daniele G., Lunghi C., Dardano A., Binda P., Ceccarini G., Santini F., Giusti L., Ciccarone A., Bellini R., Moretto C. (2021). Bariatric surgery restores visual cortical plasticity in nondiabetic subjects with obesity. Int. J. Obes..

[164] Lunghi C., Daniele G., Binda P., Dardano A., Ceccarini G., Santini F., Del Prato S., Morrone M.C. (2019). Altered Visual Plasticity in Morbidly Obese Subjects. iScience.

[165] Sui S.X., Ridding M.C., Hordacre B. (2020). Obesity is Associated with Reduced Plasticity of the Human Motor Cortex. Brain Sci..

[166] Hwang L.L., Wang C.H., Li T.L., Chang S.D., Lin L.C., Chen C.P., Chen C.T., Liang K.C., Ho I.K., Yang W.S. (2010). Sex differences in high-fat diet-induced obesity, metabolic alterations and learning, and synaptic plasticity deficits in mice. Obesity.

[167] Al-Dalaeen A., Al-Domi H. (2022). Does obesity put your brain at risk?. Diabetes Metab. Syndr..

[168] Ji Z., Wu S., Xu Y., Qi J., Su X., Shen L. (2019). Obesity Promotes EAE Through IL-6 and CCL-2-Mediated T Cells Infiltration. Front. Immunol..

[169] Bassi M.S., Iezzi E., Buttari F., Gilio L., Simonelli I., Carbone F., Micillo T., De Rosa V., Sica F., Furlan R. (2020). Obesity worsens central inflammation and disability in multiple sclerosis. Mult. Scler. J..

[170] Correale J., Marrodan M. (2022). Multiple sclerosis and obesity: The role of adipokines. Front. Immunol..

[171] Davanzo G.G., Castro G., Monteiro L.d.B., Castelucci B.G., Jaccomo V.H., da Silva F.C., Marques A.M., Francelin C., de Campos B.B., de Aguiar C.F. (2023). Obesity increases blood-brain barrier permeability and aggravates the mouse model of multiple sclerosis. Mult. Scler. Relat. Disord..

[172] Shahi S.K., Ghimire S., Lehman P., Mangalam A.K. (2022). Obesity induced gut dysbiosis contributes to disease severity in an animal model of multiple sclerosis. Front. Immunol..

[173] Spielman L.J., Little J.P., Klegeris A. (2014). Inflammation and insulin/IGF-1 resistance as the possible link between obesity and neurodegeneration. J. Neuroimmunol..

[174] Mokhtarzade M., Agha-Alinejad H., Motl R.W., Negaresh R., Baker J.S., Zimmer P. (2019). Weight control and physical exercise in people with multiple sclerosis: Current knowledge and future perspectives. Complement. Ther. Med..

[175] Bruce J.M., Cozart J.S., Shook R.P., Ruppen S., Siengsukon C., Simon S., Befort C., Lynch S., Mahmoud R., Drees B. (2021). Modifying Diet and Exercise in MS (MoDEMS): Study design and protocol for a telehealth weight loss intervention for adults with obesity & Multiple Sclerosis. Contemp. Clin. Trials.

[176] Bruce J.M., Cozart J.S., Shook R.P., Befort C., Siengsukon C.F., Simon S., Lynch S.G., Mahmoud R., Drees B., Posson P. (2023). Modifying diet and exercise in multiple sclerosis (MoDEMS): A randomized controlled trial for behavioral weight loss in adults with multiple sclerosis and obesity. Mult. Scler. J..

[177] Stenberg E., Forsberg L., Hedström A., Hillert J., Näslund E. (2021). Bariatric and metabolic surgery in populations with morbid obesity and multiple sclerosis—A nationwide, matched cohort study. Surg. Obes. Relat. Dis..

[178] Bencsath K., Jammoul A., Aminian A., Shimizu H., Fisher C.J., Schauer P.R., Rae-Grant A., Brethauer S.A. (2017). Outcomes of Bariatric Surgery in Morbidly Obese Populations with Multiple Sclerosis. J. Obes..

[179] Morales-Suarez-Varela M., Collado Sánchez E., Peraita-Costa I., Llopis-Morales A., Soriano J.M. (2021). Intermittent Fasting and the Possible Benefits in Obesity, Diabetes, and Multiple Sclerosis: A Systematic Review of Randomized Clinical Trials. Nutrients.

[180] Allogmanny S., Probst Y. (2024). Dietary Modification Combined with Nutrition Education and Counseling for Metabolic Comorbidities in Multiple Sclerosis: Implications for Clinical Practice and Research. Curr. Nutr. Rep..

